# IL-17 Is a Key Regulator of Mucin-Galectin-3 Interactions in Asthma

**DOI:** 10.1155/2021/9997625

**Published:** 2021-06-09

**Authors:** Manoj J. Mammen, Jamil Ali, Amita Aurora, Umesh C. Sharma, Ravikumar Aalinkeel, Supriya D. Mahajan, Mark Sands, Stanley A. Schwartz

**Affiliations:** ^1^Division of Pulmonary, Critical Care & Sleep Medicine, Department of Medicine, State University of New York at Buffalo, 875 Ellicott Street, Buffalo, NY 14203, USA; ^2^Division of Allergy, Immunology & Rheumatology, Department of Medicine, State University of New York at Buffalo, 875 Ellicott Street, Buffalo, NY 14203, USA; ^3^Division of Cardiology, Department of Medicine, 875 Ellicott Street, Buffalo, NY 14203, USA; ^4^WNY VA Healthcare System, Buffalo, NY 14215, USA

## Abstract

Mucus hypersecretion and chronic airway inflammation are standard characteristics of several airway diseases, such as chronic obstructive pulmonary disease and asthma. Increased mucus secretion from increased mucin gene expression in the airway epithelium is associated with poor prognosis and mortality. We previously showed that the absence of tissue inhibitor of metalloproteinase 1 (TIMP-1) enhances lung inflammation, airway hyperreactivity, and lung remodeling in asthma in an ovalbumin (OVA) asthma model of TIMP-1 knockout (TIMPKO) mice as compared to wild-type (WT) controls and mediated by increased galectin-3 (Gal-3) levels. Additionally, we have shown that in the lung epithelial cell line A549, Gal-3 inhibition increases interleukin-17 (IL-17) levels, leading to increased mucin expression in the airway epithelium. Therefore, in the current study, we further examined the relationship between Gal-3 and the production of IL-17-axis cytokines and critical members of the mucin family in the murine TIMPKO asthma model and the lung epithelium cell line A549. While Gal-3 may regulate a Th_1_/Th_2_ response, IL-17 could stimulate the mucin genes, MUC5B and MUC5AC. Gal-3 and IL-17 interactions induce mucus expression in OVA-sensitized mice. We conclude that Gal-3 may play an essential role in the pathogenesis of asthma, and modulation of Gal-3 may prove helpful in the treatment of this disease.

## 1. Introduction

Mucus hypersecretion and chronic airway inflammation are typical characteristics of obstructive airway diseases, such as chronic obstructive pulmonary disease and asthma [1]. Increased mucus secretion results from increased gene expression of the mucin family associated with increased goblet cell metaplasia in the airway epithelium [2]. Previous investigations of the mucin family's gene expression emphasized T helper type-2 (Th2) cytokines because of their pathophysiological role in allergic asthma [3]. We previously presented that the absence of TIMP-1 enhances allergic lung inflammation, airway hyperreactivity (AHR), and lung remodeling in asthma in an ovalbumin (OVA) asthma model of TIMP-1 knockout (TIMPKO) mice compared to wild-type (WT) controls [4]. We had revealed that a TIMPKO murine allergic asthma model exhibits an asthma phenotype, with airway hyperreactivity, enhanced eosinophilic inflammation, and TH_2_ cytokine gene and protein expression following sensitization with ovalbumin. [4, 5]

In a prior study, we compared the expression of several members of the galectin family and other critical cytokines in a murine allergic asthma model utilizing wild-type and TIMPKO mice [5]. We observed a significant increase in galectin-3 (Gal-3), interleukin-17 (IL-17), and transforming growth factor-*β*1 (TGF-*β*1) gene expression in lung tissue isolated from an allergic asthma murine model utilizing TIMPKO. Gal-3 gene and protein expression levels were also significantly higher in lung tissue from an allergic asthma murine model using TIMPKO [5]. We hypothesized that IL-17 provokes pulmonary neutrophilia and magnifies antigen-induced allergic response leading to increased mucus expression in OVA-sensitized mice, based on the premise that increased airway IL-17 stimulates mucin gene expression in asthmatic airways [6]. The goal of the current study was to examine the gene and protein expression levels of several key members of the mucin family, namely, MUC5AC and MUC5B, in the lung tissue obtained from the OVA-sensitized TIMPKO mice and compare them to levels in the WT controls. Additionally, we also explored the effect of Gal-3 regulation of mucin gene expression using a specific Gal-3 inhibitor peptide (Ac-SDKP) in lung epithelial cells A549 to evaluate mucin gene expression, MUC5B, and MUC5AC.

We found that lung tissue from OVA-sensitized TIMPKO mice compared to the WT controls has increased Gal-3 expression. In contrast, inhibition using a Gal-3 inhibitor resulted in a significant increase in MUC5AC and MUC5B gene expression in lung epithelial cells A549. Thus, we speculate that Gal-3 can interact with a member of the mucin family to alter cell surface polarization, increase growth factor signaling pathways, and thereby modulate lung host defense.

## 2. Material and Methods

### 2.1. Animal Model

We utilized C57/BL6 TIMPKO and WT mice that were both SHAM and OVA-sensitized as previously described. [4] Institutional Animal Care and Use Committees of the University at Buffalo and Veterans Administration Health Care System of Western New York approved animal protocols. A total of *n* = 6 samples/group were obtained.

### 2.2. Cell Line

A549 (ATCC® CCL-185™) lung epithelial cells were obtained from ATCC (Manassas, VA) and are grown as adherent cultures in complete media DMEM (Invitrogen, Grand Island, NY, USA), supplemented with 10% (*v*/*v*) fetal bovine serum (Hyclone, Logan, UT, USA), 100 units/mL penicillin, and 100 units/mL streptomycin (GIBCO, Grand Island, NY, USA). All cells were maintained in a humidified incubator with 5% CO_2_ at 37°C. The A549 lung epithelial cells were seeded into a six-well plate and treated with the Gal-3 inhibitor (Ac-SDKP) at 10 nmol concentration.

### 2.3. RNA Extraction

Cytoplasmic RNA from A549 cells was extracted using Trizol reagent (Invitrogen-Life Technologies, Carlsbad, CA) and quantitated using a Nano-Drop ND-1000 spectrophotometer (Nano-Drop™, Wilmington, DE).

### 2.4. Real-Time Quantitative PCR (QPCR)

Gene expression studies were performed on lung tissue and A549 cells (1 × 10^5^ cells/mL media) treated with and without the Gal-3 inhibitor (Ac-SDKP) (10 nmol). Relative abundances of MUC5AC, MUC5B, IL-17A, IL-17B, IL-17E, and NF-*κβ* mRNA were quantified using real-time PCR with untreated cells that were used as controls for the cell line. Gene expression levels were expressed as the relative abundance of a PCR amplified transcript via the transcript accumulation index (TAI) using the comparative CT method, as previously described. [5, 7] 500 ng of total RNA was used for the reverse transcription reaction (25 *μ*L total volume) with the first-strand cDNA synthesis kit (GE Healthcare, Piscataway, NJ), according to the manufacturer's instruction. qPCR was done using the Azuraview TM GreenFast qPCR Blue Mix LR (Azura Genomics Inc.), and well-validated PCR primer sequences were obtained from www.realtimeprimers.com. The housekeeping gene, *β*-actin, was used as an internal control. The final primer concentration used in the PCR was 0.1 *μ*M. PCR was performed using a qPCR machine (Mx3005P, Stratagene, La Jolla, CA).

### 2.5. Immunofluorescence Staining of Lung Tissue of TIMP-1 K/O and WT Mice

Standard immunofluorescent staining procedures were followed. Sections were stained using rabbit anti-mouse MUC1, MUC2, MUC4, MUC5B, MUC5AC, IL-17A, IL-17E, and IL-17B primary antibodies followed by staining with an Alexa Fluor 488-labeled anti-rabbit secondary antibody. DAPI was used as a nuclear stain. Sections were observed and photographed with a Zeiss microscope (Carl Zeiss, Oberkochen, Germany). The gene expression levels were quantified based on the intensity of the fluorescent signal analyzed using the Image J software [8] to normalize the Flour-488 (F488) intensity against the DAPI fluorescent intensity.

### 2.6. Statistical Analysis

Data are expressed as mean ± SD. Statistical comparisons were made between genotypes using a two-tailed *T*-test. A *p* value of <0.05 was considered statistically significant. Analyses were performed using Prism (GraphPad Software, Inc., San Diego, CA) software.

## 3. Results

### 3.1. Secreted Mucin Expression Levels

**Figure 1:**
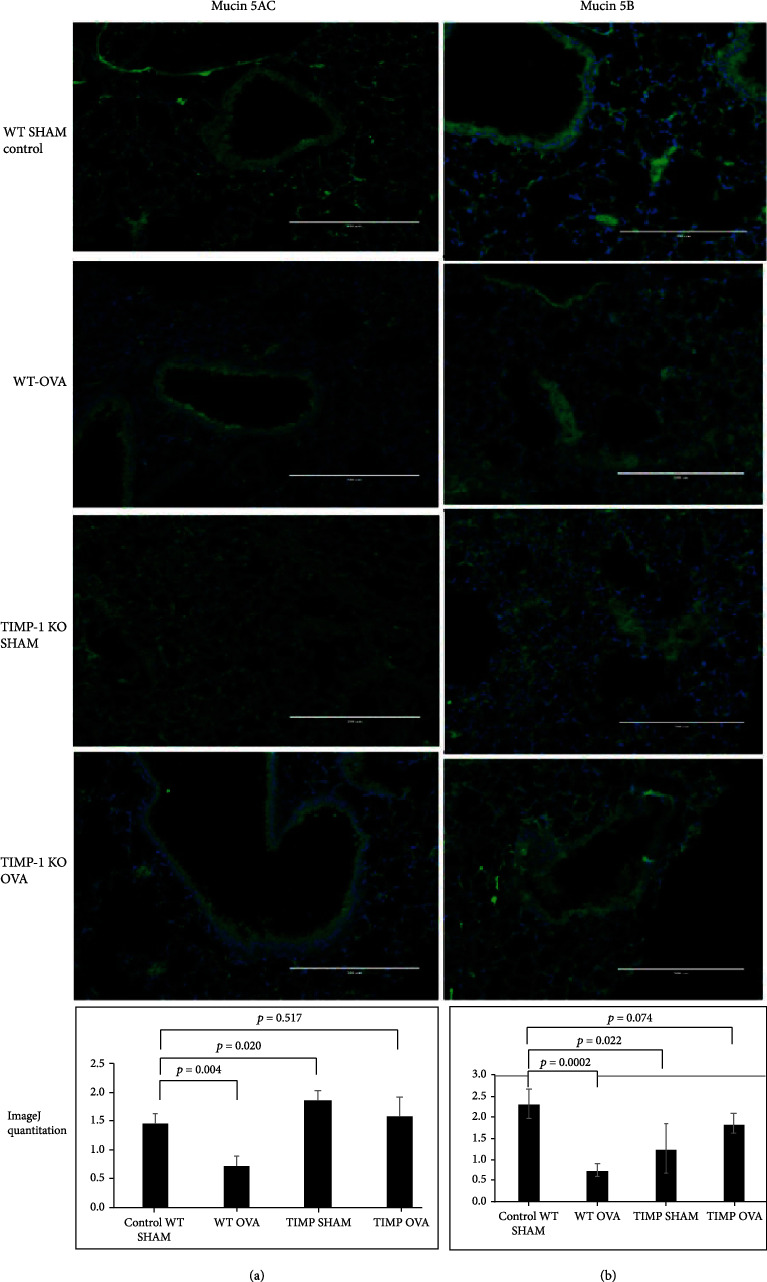
Secreted mucins MUC5AC and MUC5B expression in lung tissue obtained OVA-sensitized TIMPKO, OVA-sensitized TIMPKO, OVA-sensitized WT, and WT-SHAM mice. Representative images of immunofluorescent staining of MUC5AC (a) and MUC5B (b). The fluorescent intensity is quantitated using the ImageJ software, and the representative histogram results from data obtained from *n* = 3 separate experiments with WT-SHAM used as control.

Mucins are predominantly produced by epithelial cells on the luminal surface, and their type and quantity vary depending on location [9]. Mucins are classified into two groups: secreted and membrane-bound with MUC5AC and MUC5B, the most prevalent secreted mucins located in lung tissues. These two mucin proteins make up the majority (90%) of the mucin content of sputum, and the membrane-bound mucins (MUC1, MUC4, and MUC16) making up the remaining 10% [10]. We employed immunofluorescent staining to understand the expression profile of critical mucins better. We compared the mucin expression profile between OVA-sensitized WT (Group II), TIMPKO SHAM (Group III), OVA-sensitized TIMPKO-OVA (Group IV), and WT-SHAM (Group I) (Figure 1). There was a significant increase in the MUC5AC (133% increase; *p* = 0.020) with a significant reduction in MUC5B expression (53% decrease; *p* < 0.003) between TIMPKO SHAM (Group III) compared to the WT-SHAM control (Group I) (Figure 1).

**Figure 2:**
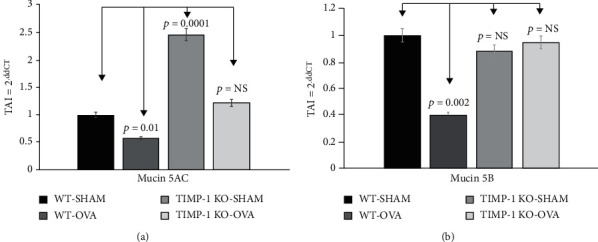
Secreted mucins MUC5AC and MUC5B gene expression levels in lung tissue obtained from OVA-sensitized TIMPKO, OVA-sensitized TIMPKO, OVA-sensitized WT, and WT-SHAM mice. Comparative real-time qPCR analysis showing relative gene expression of MUC5AC and MUC5B in lung tissue of OVA-sensitized TIMPKO, OVA-sensitized TIMPKO, OVA-sensitized WT, and WT-SHAM mice. Data normalized to the housekeeping gene *β*-actin and with WT-SHAM as control. Gene expression was calculated using the comparative CT method. Results are expressed as the mean ± SD, *n* = 3 separate experiments.

Additionally, we explored mucin family gene expression levels, specifically MUC5AC and MUC5B, that are actively secreted in the OVA-sensitized TIMPKO mouse lungs and compared them to levels in the WT-SHAM mouse lungs (Figure 2). We evaluated the MUC5AC and MUC5B gene expression in murine lung tissue obtained from the four study groups: WT-OVA (Group I), WT-SHAM (Group II), TIMPKO-SHAM (Group III), and TIMPKO-SHAM (Group IV). The gene expression levels of MUC5AC and MUC5B were quantitated using real-time qPCR. There was an increase (Figure 2(a)) in MUC5AC expression levels in TIMPKO-SHAM (Group III) (TAI = 2.46 ± 0.24; *p* = 0.0001) and TIMPKO-OVA (Group IV) (TAI = 1.22 ± 0.19; *p* = 0.173; NS) mice compared to the WT-SHAM (TAI = 1.00 ± 0.13) mice. A significant decrease in MUC5AC gene expression was observed in WT-OVA (TAI = 0.578 ± 0.09; *p* = 0.010) mice compared to WT-SHAM (TAI = 1.00 ± 0.13) mice. Our results (Figure 2(b)) show a significant decrease in MUC5B gene expression in WT-OVA (TAI = 0.406 ± 0.06; *p* = 0.002) mice as compared to WT-SHAM (TAI = 1.00 ± 0.10) mice. However, there was no significant difference in MUC5B expression levels in TIMPKO-SHAM (Group III) (TAI = 0.882 ± 0.11; *p* = 0.296; NS) and TIMPKO-OVA (Group IV) (TAI = 0.94 ± 0.098; *p* = 0.558, NS) mice compared to the WT-SHAM (TAI = 1.00 ± 0.13) mice.

### 3.2. Membrane-Bound Mucin Expression Levels

**Figure 3:**
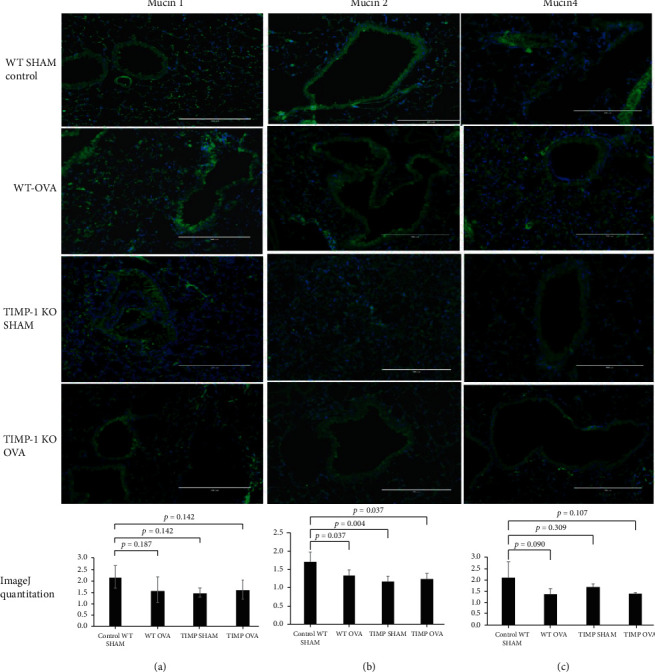
Membrane-bound mucins MUC1, MUC 2, and MUC4 expression in lung tissue obtained from OVA-sensitized TIMPKO, OVA-sensitized TIMPKO, OVA-sensitized WT, and WT-SHAM mice. Representative images of immunofluorescent staining of MUC1 (a), MUC2 (b), and MUC4 (c). The fluorescent intensity was quantitively assessed via the ImageJ software, with the representative histogram describing the data obtained from *n* = 3 separate experiments with WT-SHAM control.

Comparison of the membrane-bound mucins MUC1, MUC2, and MUC4 in the four study groups showed no significant differences in the MUC1 and MUC4 expression levels. However, we observed a significant decrease in MUC2 expression in WT-OVA (Group II) (34% decrease; *p* = 0.037), TIMPKO-SHAM (Group III) (30% decrease; *p* = 0.004), and TIMPKO-OVA (Group IV) (32% decrease; *p* = 0.037) compared to the WT-SHAM control (Group I) (Figure 3).

**Figure 4:**
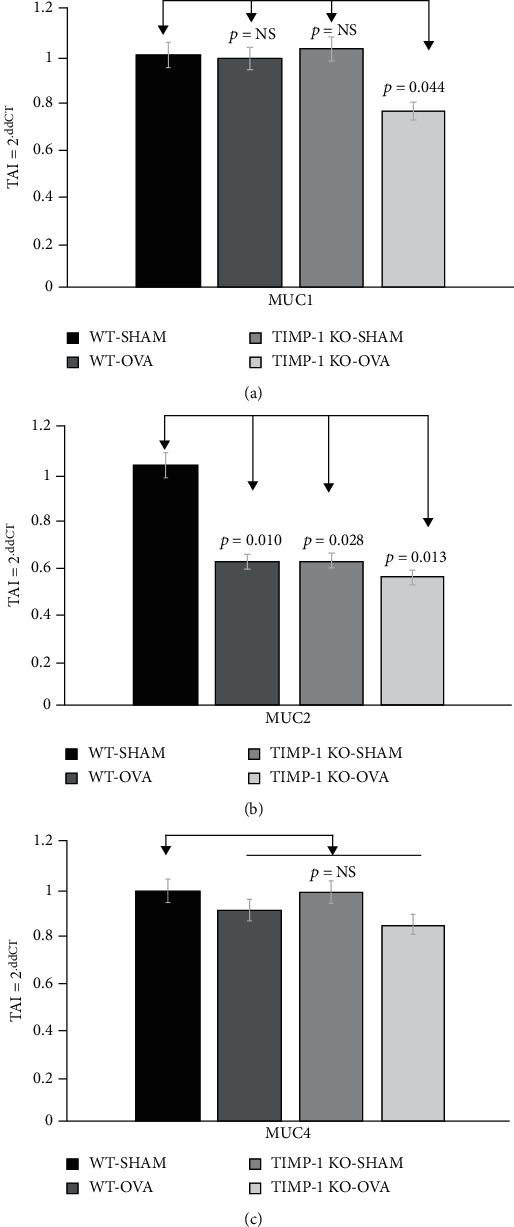
Membrane-bound mucins MUC1, MUC2, and MUC4 gene expression in lung tissue obtained from OVA-sensitized TIMPKO, OVA-sensitized TIMPKO, OVA-sensitized WT, and WT-SHAM mice. Comparative real-time qPCR analysis showing the relative gene expression of MUC1, MUC2, and MUC4 in lung tissue obtained from OVA-sensitized TIMPKO, OVA-sensitized TIMPKO, OVA-sensitized WT, and WT-SHAM mice. Data normalized to the housekeeping gene *β*-actin and with WT-SHAM as control. Gene expression was calculated using the comparative CT method. Results are expressed as the mean ± SD, *n* = 3 separate experiments.

Our results (Figure 4(b)) show a significant decrease in MUC2 gene expression in the WT-OVA (Group II) (TAI = 0.59 ± 0.12; *p* = 0.010), TIMPKO-SHAM (Group III) (TAI = 0.60 ± 0.18; *p* = 0.028), and TIMPKO-OVA (Group IV) (TAI = 0.53 ± 0.16; *p* = 0.013) mice compared to the WT-SHAM (TAI = 1.00 ± 0.11) mice. Although a small decrease in MUC1 and MUC4 expression levels was observed in WT-OVA (Group II), TIMPKO-SHAM (Group III), and TIMPKO-OVA (Group IV) mice compared to the WT-SHAM mice, no statistically significant differences in the MUC1 and MUC4 gene expression levels were observed (Figures 4(a) and 4(c)).

### 3.3. Expression Levels of IL-17A, IL-17B, and IL-17E Family Members

**Figure 5:**
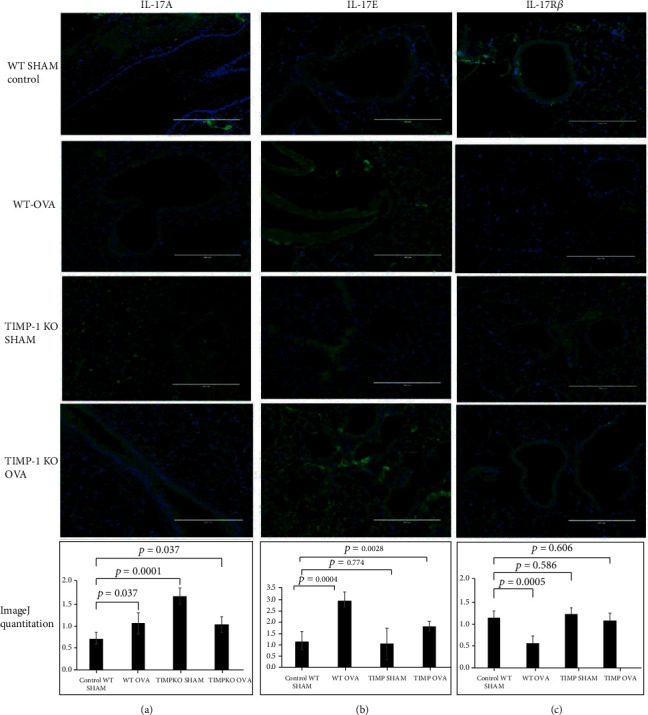
IL-17A, IL-17R*β*, and IL-17E expression in lung tissue obtained OVA-sensitized TIMP-1 K/O, OVA-sensitized TIMPKO, OVA-sensitized WT, and WT-SHAM mice. Representative images of immunofluorescent staining of IL-17A (a), IL-17E (b), and IL-17R*β* (c). The fluorescent intensity is quantitated using the ImageJ software, and the representative histogram derived from *n* = 3 separate experiments with WT-SHAM as control.

We utilized immunofluorescent staining to elucidate the expression profile variations of IL-17A, IL-17B, and IL-17E between OVA-sensitized WT (Group II), TIMPKO-SHAM (Group III), OVA-sensitized TIMPKO-OVA (Group IV), and WT-SHAM (Group I) (Figure 5). Our data (a) showed a significant increase in the IL-17A (a) expression between WT-SHAM vs. WT-OVA (42% increase; *p* = 0.037), TIMPKO-SHAM (Group III) (60% increase; *p* = 0.001), and TIMPKO-OVA (Group IV) (40% increase; *p* = 0.037) (Figure 5). As seen in (b), we observed a significant increase in IL-17E expression in WT-OVA (Group II) (260% increase; *p* = 0.0004) and the TIMPKO-OVA (Group IV) (166% increase; *p* = 0.0028) when compared to the WT-SHAM group control group. We did not observe any difference in IL-17E expression between the TIMPKO-SHAM (Group III) vs. the WT-SHAM control group (Group I). As seen in (c), we observed a significant decrease in IL-17R*β* expression in the WT-OVA (Group II) (67% decrease; *p* = 0.0005) and the WT-SHAM group control group. We did not observe any difference in IL-17R*β* expression between the TIMPKO-OVA (Group IV), TIMPKO-Sham (Group III) vs. the WT-SHAM control group (Group I).

### 3.4. IL-17 Gene Expression in TIMPKO Mice in Comparison with WT-SHAM Mice

Previous studies examining the expression of IL-17A and IL-17F in asthmatic patients' airways revealed an increase in IL-17A and IL-17F lung immunoreactivity noted in tissues obtained via bronchoscopy and showed increased IL-17A and IL-17F mRNA expression with increased asthma severity [11, 12]. IL-17A interacts with various immune factors to contribute to lung remodeling in TH_17_-driven endotypes of severe asthma. We thus profiled the IL-17A, IL-17B, and IL-17E gene expression in murine lung tissue in the four study groups: WT OVA (Group I), WT-SHAM (Group II), TIMPKO-SHAM (Group III), and TIMPKO-SHAM (Group IV).

**Figure 6:**
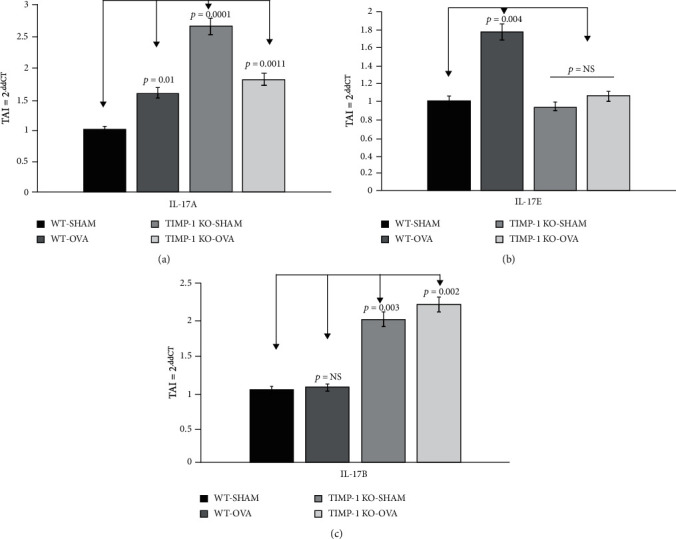
IL-17A, IL-17R*β*, and IL-17E gene expression in lung tissue obtained from OVA-sensitized TIMPKO, OVA-sensitized TIMPKO, OVA-sensitized WT, and WT-SHAM mice. Comparative real-time qPCR analysis showing relative gene expression of IL-17A, IL-17R*β*, and IL-17E in lung tissue obtained from OVA-sensitized TIMPKO, OVA-sensitized TIMPKO, OVA-sensitized WT, and WT-SHAM mice. Data normalized to the housekeeping gene *β*-actin and with WT-SHAM as control. Gene expression was calculated using the comparative CT method. Results expressed as the mean ± SD, *n* = 3 separate experiments.

Gene expression profiles of IL-17A, IL-17B, and IL-17E were quantitated using qPCR. Our results (Figure 6) showed an increase in IL-17A (Figure 6(a)) gene expression levels in the TIMPKO-SHAM (Group III) (TAI = 2.69 ± 0.30; *p* = 0.0001), TIMPKO-OVA (Group IV) (TAI = 1.82 ± 0.32; *p* = 0.011), and WT-OVA (TAI = 1.58 ± 0.11; *p* = 0.011) mice compared to the WT-SHAM (TAI = 1.00 ± 0.10) mice. Our results (Figure 6) revealed a significant increase in IL-17E (Figure 6(b)) gene expression in WT-OVA (TAI = 1.77 ± 0.18; *p* = 0.004) mice compared to WT-SHAM (TAI = 1.00 ± 0.14) mice. However, there was no significant difference in IL-17E expression levels in the TIMPKO-SHAM (Group III) (TAI = 0.939 ± 0.20; *p* = 0.643; NS) and TIMPKO-OVA (Group IV) (TAI = 1.058 ± 0.18; *p* = 0.723; NS) mice as compared to the WT-SHAM (TAI = 1.00 ± 0.14) mice. Further, our results (Figure 6) showed no change in IL-17B (Figure 6(c)) gene expression in WT-OVA (TAI = 1.04 ± 0.16; *p* = 0.772; NS) as compared to the WT-SHAM (TAI = 1.00 ± 0.15) group. However, there was a significant increase in IL-17B expression levels in the TIMPKO-SHAM (Group III) (TAI = 1.98 ± 0.28; *p* = 0.003) and TIMPKO-OVA (Group IV) (TAI = 2.18 ± 0.22; *p* = 0.002) groups compared to WT-SHAM (TAI = 1.00 ± 0.15).

### 3.5. Effect of Gal-3 Inhibitor Peptide Ac-SDKP on the Expression of MUC5AC and MUC5B Gene Expression

**Figure 7:**
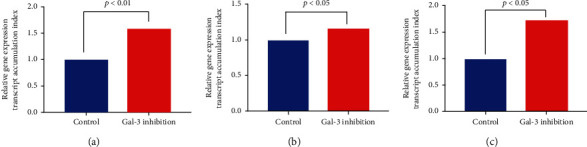
Effect of galectin-3 inhibition on gene expression of MUC5AC and MUC5B NF-*κβ* in lung epithelial cell line A549. A549 cells incubated with Gal-3 inhibitor (Ac-SDKP), and gene expression of MUC5AC and MUC5B and NF-*κβ* assessed using real-time qPCR. Comparative real-time qPCR analysis showing relative gene expression of MUC5AC and MUC5B and NF-*κβ* in A549 cells. Data normalized to the housekeeping gene *β*-actin and untreated cells were used as control. Gene expression was calculated using the comparative CT method. Results are expressed as the mean ± SD, *n* = 3 separate experiments.

We appraised the Gal-3 inhibition on the MUC5AC and MUC5B genes' expression by treating A549 cells with the Gal-3 inhibitor peptide Ac-SDKP (10 nmol). Our results (Figure 7(a)) revealed treatment with Gal-3 inhibitor peptide resulted in a 58% increase (*p* < 0.01) in MUC5AC gene expression and a 16% increase (*p* < 0.05) in MUC5B gene expression levels compared to the respective untreated controls (Figure 7(b)). Inhibition of Gal-3 results in increased MUC5AC and MUC5B in lung epithelial cells.

### 3.6. Effect of Gal-3 Inhibitor Peptide Ac-SDKP on the Expression of NF-*κβ* Gene Expression

NF-*κβ* is a ubiquitous transcription factor activated following the phosphorylation (catalyzed by I*κβ* kinase) and dissociation of its inhibitor kappa-B subunit alpha (I*κβ*-*α*). NF-*κβ*-induction is critical to the development of pulmonary inflammation [13]. Therefore, we examined the NF-*κβ* gene expression profile in the lung epithelial cell line A549 treated with 10 nmol of peptide Ac-SDKP. A Gal-3 inhibitor since NF-*κβ* signaling is critical to allergic asthma-related pulmonary inflammation. Our results (Figure 7) reveal that inhibition of Gal-3 leads to a 73% increase (*p* < 0.05) in NF-*κβ* gene expression levels as compared to the untreated control (Figure 7(c)).

## 4. Discussion

Mucus is one of the most conserved elements of host defense [14, 15]. It is comprised of a complex assortment of water, ions, proteins, glycoproteins, and lipids. Mucus is a vital innate defense component of the respiratory tract [9]. Mucins are glycosylated proteins forming macromolecular constituents of mucus and are responsible for mucus' chemical and physical properties [15]. Mucin also participates in cell signaling by interacting with immune cell receptors [10].

Predominantly produced by epithelial cells, mucins have eight members expressed in the respiratory system [15]. Mucins can be categorized into either secreted and membrane-bound, with the prevailing secreted mucins in lung tissue that are MUC5AC and MUC5B, making the majority (90%) of the mucin content of sputum [16, 17].

MUC5B is also the principal mucin in the mucociliary mucus gel layer and creates a network structure that traps debris [9]. Allergic inflammation altering the MUC5B and MUC5AC expression profiles modulates the immune system [17]. It is known that TIMP-1 binds and inactivates matrix metalloproteinases (MMP), specifically MMP-2 and MMP-9, with imbalances of the MMP-9/TIMP-1 interactions posited in the development of asthma [18–21]. Additionally, MMP9 activity modulates MUC5AC production via the EGFR pathway [22, 23].

In our study, we examined the expression levels of critical members of the mucin family, specifically the secreted mucins MUC5AC and MUC5B, in addition to the membrane-bound mucins (MUC1, MUC4, and MUC2) in lung tissue obtained from the TIMPKO murine asthma model. We have previously published that OVA-treated TIMPKO mice develop an asthma phenotype data with airway hyperreactivity [4]. Additionally, we have shown that increased expression of Gal-3 protein in OVA-treated TIMPKO mice leads to increased inflammatory mediators such as TGF-*β*1 and IL-17 that exacerbate asthma [5]. We postulated that IL-17 augments the antigen-induced allergic response, provokes pulmonary neutrophilia, and increases mucus expression in OVA-sensitized mice. This hypothesis is based on the premise that increased lung levels of IL-17 stimulate mucin gene expression in asthmatic airways [6]. The goal of the current study was to examine the gene and protein expression levels of several key members of the mucin family, namely, MUC5AC and MUC5B and MUC1, MUC2, and MUC4 in the lung tissue obtained from the OVA-sensitized TIMPKO mice and compared them to levels in the WT controls.

TH_17_ cell-derived cytokines and factors appear to mediate airway neutrophilia in severe asthma [11]. Additionally, TH_17_-secreted IL-17A impacts airway smooth muscle (ASM) remodeling in severe asthma [12]. TH_17_-derived cytokines and diverse immune mediators modulate ASM functionality by interacting with airways' structural cells [24, 25]. And there appears increased IL-17A and IL-17F mRNA expression with increased asthma severity [11, 12]. We evaluated the IL-17A, IL-17B, and IL-17E gene expression in lung tissue obtained from mice in the four study groups: WT OVA (Group I), WT-SHAM (Group II), TIMPKO-SHAM (Group III), and TIMPKO-SHAM (Group IV). Our results (Figure 5) showed an increase in IL-17A and IL-17B expression levels in TIMPKO-OVA compared to the WT-SHAM mice. However, there was no significant difference in IL-17E expression levels in TIMPKO-OVA than in the WT-SHAM. Our previous study showed increased TGF-*β*1 levels in TIMPKO mice, and we believe that it may be an essential mediator of TH_17_ differentiation and IL-17A production [5]. Thus, we speculate that IL-17A and IL-17B interact with various immune factors, specifically TGF-*β*1, to contribute severity of asthma.

Gal-3 is a *β*-galactoside-binding lectin with various functions, including the regulation of Th1 and Th2 responses [26–28]. As indicated in studies above and this study, TH_17_ cells may be important mediators of lung tissue inflammation and contribute to the lung pathophysiological processes that contribute to asthma progression, and Gal-3 may affect the IL-17 expression profile in asthma [29–31]. Studies have shown that Gal-3 inhibits the Th2 allergic asthma; however, it is important to recognize that the role of Gal-3 in Th1/Th2 immune and inflammatory responses may vary according to the experimental models used to study allergic asthma. Gal-3 inhibition could inhibit Th2 response and may have a valuable therapeutic role in allergic asthma [2, 32].

NF-*κβ* plays a key role in IL-17A signaling cascades as evidenced by its stimulation of the degradation of I*κβ*-*α*, followed by the nuclear translocation of p50 and p65 subunits of NF-*κβ* and induction of mucin gene expression, thus suggesting that NF-*κβ*-based transcriptional mechanisms are involved in the regulation of mucins via IL-17A activation in the airway epithelium [33, 34].

Increased Gal-3 in mice with TIMPKO, murine asthma model is associated with decreased MUC5AC and MUC5B levels, while inhibition of Gal-3 results in increased MUC5AC and MUC5B in a lung epithelial cell line. The galectin known to associate with mucins is Gal-3, which interacts with MUC1, MUC4, and MUC16 to alter cell surface polarization and increase growth factor signaling pathways [14, 35]. Gal-3 appears to be overexpressed in fibrotic tissue, indicating a possible role for Gal-3-directed therapeutics in fibrotic lung disease [36, 37]. While the precise mechanism detailing Gal-3 association with fibrosis is unknown, and evidence suggests Gal-3 stimulates fibroblasts and TGF-*β*1-mediated signaling in vitro [37]. We have previously demonstrated TGF-1*β* activation in the lung tissue of TIMPKO mice [5]. In this study, we treated lung epithelial cells A549 with Gal-3 inhibitor and observed a significant increase in MUC5AC and MUC5B (Figure 7), corroborating the prior studies by Piyush et al. [35].

Furthermore, we report an increase in secreted mucins vs. decreased or no change in membrane-bound mucin expression levels in lung tissue of TIMPKO mice as compared to the WT-SHAM mice indicating that the secreted mucins MUC5AC and MUC5B may be more critical to airway defense and asthma disease progression compared to the membrane-bound mucins that may play a significant role in lung fibrosis and tumor progression. Further changes in the levels of secreted mucins may change the mucus barrier's physical properties, thereby modulating the immune response. Elucidating mucins' role in modulating the asthmatic immune response should identify novel therapeutic approaches for airway inflammation in asthma. Our data suggest that Gal-3 inhibition increases IL-17 levels, increasing mucins' expression, and potentially, a role for NF-*κβ* in the Gal-3 and IL-17 mediated MUC5AC and MUC5B regulation in the airway epithelium.

While our study has many strengths, there are some limitations that warrant further study in subsequent experiments. Our TIMPKO asthma model was developed with the C57BL/6 background which is more difficult to demonstrated allergic sensitization compared to BALB/c strains [38]. The animal model we explored is an acute allergy injury model, which may not fully represent a chronic allergic asthma phenotype. Also, the TIMPKO model likely has novel developmental aberrations in the pulmonary system which would be more precisely explored in a targeted conditional knockout model. Finally, TIMP-1 knockout likely has pleiotropic effects, and a targeted knock-in and knock-out models of Gal-3 should allow a more precise understanding of Gal-3 immunomodulatory effects.

We believe that allergen-reactive Th_2_ cells and proinflammatory cytokine such as IL-17 play an essential role in the induction and continuation of the inflammatory cascade in allergic asthma. Gal-3 modulates the IL-17 axis, which regulates mucin gene expression of MUC5AC, leading to mucus hypersecretion and persistent airway inflammation in asthma.

## Data Availability

The data supporting the study findings are available from the corresponding author upon pragmatic request.

## Abbreviations

TIMP-1: Tissue inhibitors of metalloproteinase-1
ECM: Extracellular matrix
Gal: Galectin
Gal-3: Galectin-3
MMP: Matrix metalloproteinase
WT: Wild-type
OVA: Ova-albumin
ASM: Airway smooth muscle
AHR: Airway hyperreactivity
BALF: Bronchoalveolar lavage fluid
i.p.: Intraperitoneal
IL-17: Interleukin-17
TIMPKO: TIMP-1 knockout.
